# What is pesticide-induced epilepsy?

**DOI:** 10.1016/j.clinsp.2024.100515

**Published:** 2024-11-08

**Authors:** Fulvio A. Scorza, Raphael Wuo-Silva, Rozana M. Ciconelli, Josef Finsterer, Feres Chaddad-Neto

**Affiliations:** a Disciplina de Neurociência, Escola Paulista de Medicina/Universidade Federal de São Paulo (EPM/UNIFESP), São Paulo, SP, Brasil; b Unidade de Neurocirurgia, Hospital A Beneficência de São Paulo, São Paulo, SP, Brasil; c Departamento de Pesquisa da BP, A Beneficência Portuguesa de São Paulo, São Paulo, SP, Brasil; d Departamento de Neurologia e Neurocirurgia, Escola Paulista de Medicina/Universidade Federal de São Paulo (EPM/UNIFESP), São Paulo, SP, Brasil; e Neurology and Neurophysiology Center, Austria

Epilepsy is one of the most common neurological disorders affecting up to 70 million people worldwide.^1^^,^^2^ It can affect individuals of all ages, ethnicities, social classes, and geographical locations.^2^^,^^3^ It is treatable in the majority of cases but often requires lifelong medication and sometimes resective surgery, neuromodulating devices, or dietary therapies to control seizures. ^2^^,^^4^, ^5^, ^6^ Despite this, up to a third of people with epilepsy do not respond properly to antiepileptic drugs or other treatments.^2^^,^^4^, ^5^, ^6^ In these individuals with drug-resistant epilepsy, epilepsy should be considered a malignant disease, as the mortality rate is 2-3 times higher than that in the general population.^2^^,^^4^^,^^6^^,^^7^ Sudden and Unexpected Death in Epilepsy (SUDEP) is the most common type of death in people with epilepsy.^8^, ^9^, ^10^ In fact, several predisposing and precipitating factors may coexist and contribute to SUDEP, but the mechanisms are poorly understood.^8^, ^9^, ^10^ In general, cardiac dysfunction appears to play an important role in SUDEP.^8^, ^9^, ^10^ The causes of epilepsy are classified into established categories, such as genetic, structural, metabolic, infectious, immune, and unknown causes.^4^ Furthermore, other epidemiological studies have shown that specific risk factors, such as parasitic diseases (e.g., neurocysticercosis), may explain the high incidence of epilepsy in developing countries, including Brazil.^11^^,^^12^ Given the high scientific relevance of these data, it is also important to make some additional considerations that may open the debate on the extent to which human exposure to pesticides could be a possible risk factor for epilepsy and thus for SUDEP.

Brazil is one of the largest consumers of pesticides in the world.^13^ In 2020, there was a total of 83,396,004 ha of cultivated land in the studied country.^14^, ^15^, ^16^ At the same time, pesticide reports from specialized institutes showed that the amount of pesticides sold in Brazil in the same period was 685,745.68 tons, indicating that the amount of pesticides sold in Brazil has increased threefold compared to the growth in cultivated areas.^14^, ^15^, ^16^ The data presented by the National Health Surveillance Agency (ANVISA) is alarming, as it shows that of the total 504 pesticide active ingredients approved for use in Brazil, 397 were industrially produced chemicals, 146 of which are not approved in Europe and therefore cannot be marketed.^14^, ^15^, ^16^ Unfortunately, pesticides are known to cause acute toxicity when a high dose is inhaled, ingested, or comes into contact with the skin or eyes, while prolonged exposure leads to chronic toxicity.^17^ In this sense, exposure to pesticides is known to cause various types of adverse health effects, such as dermatologic, gastrointestinal, respiratory, reproductive, endocrine, child growth, carcinogenic, and neurological effects.^18^, ^19^, ^20^, ^21^ Considering the neurotoxic effects, new data suggest that exposure to some pesticides (i.e., carbamates, organochlorines and organophosphates) is a possible, but still underestimated cause of brain disorders, including epilepsy.^18^^,^^22^, ^23^, ^24^ Firstly, several animal models (nonhuman primates, rats, mice, and guinea pigs) of organophosphate-induced *status epilepticus* have contributed significantly to understanding of epileptogenesis and the mechanisms of action of new antiepileptic drugs.^25^, ^26^, ^27^ Indeed, some epileptogenic pesticides such as lindane, endosulfan, chlordimeform, amitraz, and chlorpyrifos are capable of inducing epileptic seizures after repeated exposure at low doses.^28^ Similarly, other classes of pesticides have been shown to induce seizures as side effects, including λ-cyhalothrin, fipronil, glufosinate, lufenuron and mepiquat.^28^ Importantly, the first study showing a link between long-term low-dose pesticide exposure and epilepsy was published in 2018. The authors elegantly demonstrated that the prevalence of hospital-diagnosed epilepsy was higher in populations living in areas of high pesticide use than in areas of low pesticide use.^28^ Recently, the same research group not only confirmed the link between epilepsy and pesticide exposure in the general population but also extended this risk to farmers who are occupationally exposed to pesticides, especially those who work in industrial agriculture and use no or improper personal protective equipment.^24^

In line with the following consideration could also be made: is it possible that pesticide exposure favors the occurrence of cardiovascular abnormalities, and thus SUDEP, in people with epilepsy? Yes, this is possible. Since research in this area must be oriented towards the possible cardiovascular mechanism of SUDEP,^29^ some arguments might be put forward. In fact, it has been observed that abnormally elevated levels of ingested or inhaled organophosphorus and organochloride pesticides can lead to oxidative stress and inflammation of the heart, promoting myocardial fibrosis and cardiac arrhythmias.^30^ In addition, patients hospitalized for acute pesticide poisoning often suffer from atrial fibrillation or malignant ventricular arrhythmias.^30^ Moreover, several studies clearly demonstrate a strong link between agrochemical particles and cardiovascular diseases in agricultural workers.^31^ Importantly, the authors conclude that a wide variety of pesticides used by farmers and the prevalence of unreported deaths from cardiovascular disease are a serious concerns to the healthcare industry.^31^ Since ingested or inhaled pesticides are associated with adverse cardiac effects. 30,31] and seizures often impair cardiac function,.^7^, ^8^, ^9^, ^10^^,^^29^ it is very plausible to assume that these factors together could negatively affect the cardiovascular system of epilepsy patients and lead to cardiac dysfunctions and thus SUDEP. While acknowledging that this knowledge of the exact relationship between pesticides/herbicides/insecticides ‒ including organophosphorus and organochloride compounds ‒ and epilepsy is insufficient, the present study's research group believes that new studies are needed to accurately unravel the relationship between pesticide exposure and epilepsy to minimize the population's risk.

So what happens next when you take all these data together? Firstly, the authors are firmly convinced that exposure to pesticides in the environment is associated with a higher risk of epilepsy. Secondly, depending on the amount and type of exposure, ingestion of pesticide residues in food, poses a serious risk and that should be intensively discussed in the medical community. Third, it is important to consider the consumption of organic food as it contains lower levels of potentially toxic substances. Although organic food tends to be more expensive than conventionally produced food, there are many reasons behind consumer purchase of organic products, including concerns for your health, food safety, and the environment.^32^ Finally, the most important step is to educate the population that maximizing profits is not everything and that human exposure to pesticides is directly linked to chronic diseases that can often be fatal.

## Conflicts of interest

The authors declare no conflicts of interest.
